# Proxy reporting in health: a scoping review of instructions, perspectives, and reporting experiences

**DOI:** 10.1007/s11136-025-03929-8

**Published:** 2025-02-26

**Authors:** Henok Dagne, Kathleen Doherty, Julie Campbell, Alice Saul, Jessica Roydhouse

**Affiliations:** 1 Menzies Institute for Medical Research, University of Tasmania, Hobart, TAS Australia; 2 University of Gondar, Gondar, Ethiopia; 3 Wicking Dementia Research and Education Centre, University of Tasmania, Hobart, TAS Australia

**Keywords:** Proxy, Perspective, Instruction, Experience

## Abstract

**Background:**

Informal carers (‘proxies’), who typically provide unpaid care and have a personal relationship with the care recipient, are often asked to report on the health of individuals who are unable to self-report. However, this task is not without its challenges. Little is known about how proxies approach this task, which poses challenges for optimal questionnaire design.

**Purpose:**

The review had three aims: (1) to identify the questionnaire perspective instructions provided to proxies i.e., whether the proxy was asked to answer from their own (proxy–proxy) or the person’s (proxy–patient) perspective when reporting, (2) identify which perspective instruction the proxies followed, if reported, and (3) what information, if any, was captured about the proxy’s experience of reporting about someone else. In the proxy–proxy perspective, proxies report from their own perspective, but in the proxy–patient perspective they report from the perspective of the person living with the condition.

**Methods:**

A systematic search was conducted across Medline, Psych Info, CINAHL, and Embase. Only published articles meeting the criteria of informal carers providing proxy reports for adults were considered.

**Results:**

Of 5816 publications, 60 articles were eligible for full-text review, and 12 were included for data extraction. Instructions varied, with proxies asked to adopt both proxy–proxy and proxy–patient perspectives in eight studies and only the proxy–patient in four of them. Whether proxies followed the provided instructions consistently is not known. Proxies’ reporting experiences were not reported in the included studies.

**Conclusion:**

Proxies are provided with different perspective instructions, but proxy adherence to instructions is not known. Additionally, proxy reporting experience was not described. Providing clear instructions, evaulating proxy adherence to instructions and collecting proxies’ reporting experiences can inform optimal questionnaire design to help proxies better report about the health of others.

**Review registration:**

Registered at open science framework: https://osf.io/j4t87.

**Supplementary Information:**

The online version contains supplementary material available at 10.1007/s11136-025-03929-8.

## Plain language summary

In this review, we explore the instructions provided to informal carers (‘proxies’) completing questionnaires for individuals who are unable to complete questionnaires. By understanding these instructions, informal carers can provide useful information about the heath-related quality of life and symptoms of others. This will help to improve the quality of data collected from proxies. This review identified a lack of consistency in how proxies are instructed to report on the health of people who are unable to complete questionnaires. More consistent instructions and understanding how well proxies follow instructions may be helpful.

## Background

Individuals with advanced illnesses or cognitive impairments can encounter challenges in reporting their own health status [1–3]. In such instances, proxies, often caregivers or family members, may be asked to provide this information. The use of proxy respondents is common in epidemiological research [4, 5]. Proxies are often used in specific populations such as children [6, 7], older adults [3, 8, 9],or in specific clinical contexts such as dementia [2, 10], hearing loss [11] or critical illness [12]. They are typically asked to provide their perspectives in situations where patients have cognitive impairments, severe illnesses, or are otherwise incapacitated [13].

This scoping review focuses on informal caregivers, who are typically family members, friends, or neighbours providing unpaid care. In contrast, formal caregivers are paid professionals who have received training in caregiving and may be employed at healthcare institutions or private agencies in which they provide care to individuals. We chose to focus on informal caregivers because they often lack the professional training that formal caregivers receive, which can lead to unique challenges in reporting the health-related quality of life and symptoms of the person they care for due to limited healthcare knowledge. Greater understanding of the instructions given to informal caregivers, as well as their experience of proxy reporting, can inform better questionnaire design and, ultimately, potentially better reports from informal caregivers acting as proxies. In this review we focused on quality-of-life measures including health-related quality of life and symptom measures.

Reporting on behalf of someone else presents several challenges, primarily due to proxies’ limited access to complete firsthand information compared to individuals reporting on their own health [14, 15]. Proxies may lack complete insight into an individual’s health history and symptoms, leading to difficulties in reporting. Proxies acquire information differently than individuals who self-report. While self-reporting individuals draw information from direct experiences, proxies rely on observation or communication fundamentally shaping their reporting approaches [16]. This difference can make it challenging for proxies to provide a response that closely aligns with what the individual would have reported [17]. Proxy reports are affected by a number of factors, including proxies’ perception, memory, motivation to respond, the type of information to be reported [15, 18], and how the proxy engages with the individual, including the type of relationship, and the frequency of contact [18, 19].

Proxies may be asked to report about others from their own (“proxy–proxy”) or the individual’s (“proxy–patient”) viewpoints [20, 21]. The former is often defined as proxies responding from their own experience based on their observation and interpretation, while the latter entails proxies attempting to report as if they are the patient themselves and reconstruct the patient’s internal mental state when answering questions [22–24]. In the proxy–proxy perspective, proxies are asked to respond based on their own observations and interpretations of the patient’s condition. They provide information from their viewpoint, reflecting what they see and understand about the patient’s health-related quality of life or symptoms [21, 25]. To illustrate these concepts, one might imagine a proxy being asked in the proxy–proxy perspective, as “How much pain do you think the patient is experiencing?” In contrast, from the proxy–patient perspective, a question might be phrased as, “If you were the patient, how much pain would you say you are experiencing?” The US Food and Drug Administration (FDA) [26] and the European Medicines Agency (EMA) [27] definition of proxy report uses the proxy–patient perspective. Research suggests that instructions provided to proxies regarding perspective-taking influence their responses to questionnaires [28–30]. However, whether proxies follow these instructions is not usually examined [28, 30]. There is no clear evidence indicating that one perspective may better enable proxies to provide responses similar to patient responses, with inconsistent results that vary across different health domains [30–32]. Neither proxy–proxy nor proxy–patient perspectives have been shown to have consistently better concordance with self-reports for all domains or conditions. Rather, there is some evidence of better concordance for one perspective with self-report for some domains, and for the other perspective with self-report for other domains. For example, a study in an orthopaedic outpatient clinic assessing agreement between self-completed EQ-5D-5L scores by patients and the proxy reports found substantial agreement between proxy and patient reports while using the proxy–patient perspective and moderate agreement when the proxy–proxy perspective was used [32]. Lobchuk et al. reported that asking the proxies to imagine themselves as the patients was effective in reducing discrepancies across symptoms and underlying symptom dimensions (e.g. severity, distress and frequency) [22]. However, Gundy and Aaronson found that concordance between patient and proxy reports was better for functional scales on a cancer-specific tool when using the proxy–proxy perspective [30]. A review on the effect of perspective on the report difference between the self-reported and proxy-reported quality of life of people living with dementia has indicated that adopting a proxy–patient perspective reduces the difference between proxy and patient report compared to the proxy–proxy perspective [33].

Proxy reports may influence clinical decisions, treatment plans, and interventions. If proxies provide inaccurate information and if the information is used in clinical decisions, it can impact patient care. A study indicated that it is easier for proxies to report on observable factual information than private subjective behaviour [18]. This is due to the fact that proxies may not have the same level of insight to the patient’s experience as the patients themselves. Recognising proxies’ confidence in reporting may help to judge the quality of evidence prior to using the information for decision making. Factors such as proxies’ knowledge and understanding of the health situation of the care recipient may be associated with their confidence. When proxies are confident, their reports may more likely reflect the patient’s true condition and preferences. Conversely, a lack of confidence may indicate potential uncertainties in the information provided [34]. When developing proxy-reported instruments, cognitive interviews may be used to identify potential issues in understanding and interpreting survey questions. This can help refine questions to ensure they are clear and comprehensible for proxies, thereby improving the accuracy of their responses [35]. During these cognitive interviews, proxies may be asked additional questions such as how difficult they find it to respond on behalf of someone else [36], and how confident they are that their responses align with those of the target individuals [18].

In the current scoping review, the term ‘proxy reporting experience’ refers to any question asked to proxies about their experience while completing a questionnaire for someone else including their level of confidence in their report. There is a knowledge gap regarding the specific instructions provided to proxies about perspective-taking [25, 37], the perspectives they adopt, and proxies’ experience when reporting about others [14]. Understanding whether specific instructions or prompts regarding proxy perspectives are provided to proxy respondents may be helpful. By identifying this, we can assess whether researchers and practitioners guide proxies on how to approach reporting (e.g., proxy–proxy or proxy–patient perspective). Knowing which perspective (if any) proxies adopt while completing health questionnaires may provide valuable insight into which perspectives they may find more useful. Examining how proxies’ reporting experiences are captured in published articles allows us to explore the experience of proxy reporting and understand real-world challenges. This scoping review had three specific objectives:

1. To identify whether instructions or prompts to use either of the proxy perspectives are provided in the patient health questionnaires for proxy completion by informal carers.
2. To identify which perspective, if any, was adopted by informal carers while completing patient health questionnaires about others.
3. To identify if and how proxies’ reporting experiences are captured across published articles.

This scoping review aims to shed light on the current practices in proxy reporting, including the instructions provided to proxies, the perspectives they adopt, and their experiences in reporting. By examining these aspects, we anticipate uncovering patterns in proxy use across different clinical populations and identifying potential areas for improvement in proxy assessment.

## Method or design

A scoping review was used to address the research questions. It was chosen over a systematic review as it focuses on mapping the evidence landscape and discerning key knowledge gaps in the field [38]. Unlike systematic reviews, a scoping review allows for the exploration of broad and less specific research questions [38–40]. The approach taken in this scoping review adhered to the methodological guidelines proposed by the Joanna Briggs Institute (JBI) [41]. We employed the framework outlined by Arksey and O’Malley [42], encompassing a structured process involving five key stages: (1) formulating the review questions, (2) identifying relevant studies, (3) selecting eligible studies, (4) charting and collating data, and (5) summarising and reporting results. We did not undertake the sixth optional stage of the scoping review, which is stakeholder consultation [43]. We used the Preferred Reporting Items for Systematic reviews and Meta-Analyses extension for Scoping Reviews (PRISMA- ScR) for scoping reviews to present the results [44].

### Stage 1. Formulating review questions

This was an iterative process. HD and JR developed, revised, and refined the research questions with input from KD and JC. Revisions were undertaken by reading relevant studies and checking their findings. Preliminary searches were conducted on two databases (Medline and CINAHL) to see whether the search strategies provide articles that will answer the research questions. Based on the results, the research questions were rephrased. Broad research questions were narrowed down after discussion based on the focus of the review. For example, our initial question was about instructions for proxy reporting, and we subsequently narrowed this to focus on perspective instructions. Similarly, we had an initial question on proxy experience but then narrowed this to focus on reporting experience. All these happened before the actual search for the final review. The alignment of the objectives and research questions was checked and discussed within the team. The scope and significance of the review was agreed after rounds of discussion.

### Stage 2. Identifying relevant studies (search strategy)

After formulating the research question, a search strategy was developed in consultation with university librarians (Supplementary File 1). JR and HD undertook double screening of all abstracts.

### Stage 3. Study selection

HD assessed studies for inclusion based on pre-specified Population, Concept and Context (PCC) criteria suggested by JBI [41], as indicated in Table 1. Original articles written in English were eligible if they included informal carers of adults with any health condition. The target population was informal carer of adults who typically provide unpaid care and have a personal relationship with the care recipient. The concepts were instructions provided to carers about perspectives, adherence to the instructions and reporting experience of carers. All retrieved articles were exported to Endnote 7.0 and then to Covidence (www.covidence.org, Veritas Health Innovation Ltd.).

**Table 1:** Eligibility criteria for study inclusion

Criteria	Inclusion	Exclusion
Population	Informal carers of adults	• Formal carers only
	Informal AND formal carers	• Carers acting on care plan and proxy decision making,
		• Carers acting as agent in care plan
Concept	• Proxy perspective completion instructions,	• Concepts other than the three mentioned ones
	• Proxy perspectives instruction adherence and	
	• Proxies’ reporting experience	
Study type	• Original articles reporting on either of the three concepts	• Articles that do not include any of the three concepts
	• Validation studies if any proxy instruction	• Validation studies with no information about perspectives
	• Concordance studies with multiple perspectives	• Articles written in language other than English
	• Articles written in English	• Grey literature
Time	Until 13/07/2023 (no starting date and no country-related limitations were applied),	
Study design	Primary studies with any design	
Information sources	Medline via Ovid, Psych Info, CINAHL, and Embase	
Key terms	*Proxy terms*: proxy report, external rater report, carer report, family report	
	*Perspective*: proxy version, proxy perspective, proxy–patient, proxy–proxy	
	*Quality of life term*: QOL, HRQL, HRQOL	
	Full search strategy (Supplementary File 1)	

### Stage 4. Data extraction/charting

The JBI template source of evidence details, characteristics and results extraction instrument [41] was used to extract and chart the data in Covidence. HD extracted data and charted evidence listing the study authors, year of publication, country, sample size, carers involved, health condition, and major findings. JR checked the extracted data and added comments and suggestions. Discrepancies were resolved through discussion. HD revised the data extraction based on comments from JR.

### Stage 5. Collating, summarising, and reporting results

The evidence was summarised in relation to objectives and review questions. HD wrote the report. JR, JC, AS and KD revised the report, and the final version was approved by all authors.

The scoping review methods are documented in the review protocol. The protocol is registered in the Open Science Framework [45]. The protocol was amended from the original version as follows. Initially, the focus was on proxy reports by informal carers only. However, we expanded the scope to encompass studies that included both formal and informal carers, as these studies might have provided significant insights into the experiences of informal carers. We also amended the protocol to include dyadic studies if they included information about both perspective instructions, and to include validation studies. The amendment was necessary as these issues become clearer during the review exercise (Tables 1 and S1).

## Results

A total of 5816 articles were identified, from which 2104 duplicate records were removed. Following title and abstract screening, 3652 articles were excluded, and 60 studies were retained for full-text review. Based on a full-text review, 44 studies were excluded: most of them (23 out of 44) do not address the concepts of the research questions. Initially, 16 studies were considered for data extraction. Four of the 16 studies were found to be ineligible during data extraction.

### Study characteristics

**Fig. 1:**
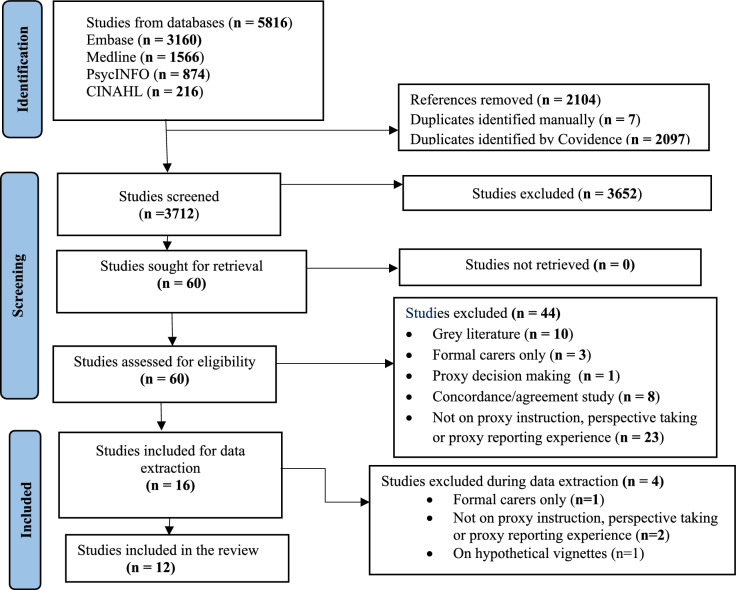
PRISMA-ScR flow chart showing the selection of studies for the review

In the final review, 12 studies [14, 22, 30, 32, 46–53] were included (Fig. 1). The aim of each included study is indicated in Table S2. Six out of the 12 studies were conducted in United Kingdom [14, 48–52]. Five of the included studies were dyadic studies in which information from both proxies and patients were captured [22, 30, 32, 46, 47]. Five of the studies were validation studies, and all of them involved carers of people living with dementia [14, 48, 51–53], and three studies were among carers of people with cancer [22, 30, 47]. There were eight studies among informal carers only [22, 30, 32, 46, 47, 51–53], and four among both informal and formal carers [14, 48–50]. Half of the studies were among carers of people living with dementia [32, 49–53] (Table 2).

**Table 2:** Summary of key characteristics of included studies in the review

Study	Country	Carer/proxy	Health condition	Study method/approach	Sample size	Measurement tool used
Caiels et al. [14]^a^	UK	Informal and formal carers	Intellectual disability	Qualitative	43 (35 paid and 8 unpaid)	ASCOT
Gundy and Aaronson [30]^b^	Netherlands	Informal carers (partners/spouses: > 70%)	Cancer	Quantitative	224	EORTC QLQ-C30
Hong et al. [46]^b^	US	Family (maternal) carers	Autism spectrum disorder	Quantitative	60	WHOQOL-BREF
Lobchuk et al. [22]^b^	Canada	Informal carers	Breast and prostate cancer	Quantitative	126	MSAS (shortened version)
Pickard et al. [47]^b^	US	Family carers	Prostate cancer	Quantitative	87	EQ-5D EORTC QLQ-C30
Rand et al. [48]^a^	UK	Informal carers or formal care workers	People with cognitive or communication impairments	Qualitative	25 (13 care workers, 12 family carers)	ASCOT
Robertson et al. [49]^c.d1^	UK	Staff and relatives	People living with dementia in care homes	Qualitative	24 (12 staff and 12 relatives)	DEMQOL-Proxy
Robertson et al. [50]^c,d1^	UK	Staff and family members	People living with dementia in care homes	Both quantitative and qualitativ	1056 (quantitative), 12 (qualitative)	DEMQOL-Proxy
Silarova et al. [51]^b,d2^	UK	Informal carers	People living with dementia	Qualitative	313	ASCOT
Smith et al. [52]^b^	UK	Family caregivers	People living with dementia	Both qualitative and quantitative	126	DEMQOL-Proxy
Stephan et al. [53]^a,d3^	Germany and Portugal	Informal carers	People living with dementia	Qualitative	23 carers (14 spouses, 8 children, 1 other)	ICECAP-O
Tol et al. [32]^b^	Netherlands	Informal carers (spouses)	Orthopaedic conditions	Quantitative	115	EQ-5D-5L

^a^Validation study

^b^Dyadic study

^c^Other study

^d^Part of larger study (1.MARQUE, 2. MOPED, 3. Artifcare)

The tools used to assess patient health in the included studies were ASCOT [14, 48, 51], EORTC QLQ-C30 [30, 47], WHOQOL-BREF [46], ICECAP-O [53], EQ-5D [32, 47], MSAS (shortened version) [22] and DEMQOL-Proxy [48, 50, 52] (Table 2). We classified the included studies as quantitative, qualitative and mixed based on the approaches used. Here, we define qualitative studies as those using techniques such as in-depth interviews or focus groups, where interpretive or other analyses may be employed. In contrast, quantitative studies use techniques for analysing numerical or quantitative data. Mixed method studies can incorporate both qualitative and quantitative approaches [54]. Using our definitions, this scoping review includes five qualitative studies [14, 48, 49, 51, 53], two mixed-method studies [50, 52] and five quantitative studies [22, 30, 32, 46, 47]. The sample size for the quantitative studies included in the review ranged from 60 to 224. The median number of the study participants for the included quantitative studies was 115 (Table 2).

#### Perspective instructions or prompts provided to proxies (1st objective)

Carers were asked to adopt different perspectives when reporting as proxies. In all five dyadic studies [22, 30, 32, 46, 47] and three of the seven non-dyadic studies [14, 48, 51] proxies were instructed to respond from both the patient’s perspective and their own perspectives (Table 3). In four of the studies [49, 50, 52, 53], proxies were asked to report only from the proxy–patient perspective. The instructions provided varied across the included studies, even for the same perspective, in their emphasis on empathy, perception of patient feelings and imagining the patient’s experience (Table S3). For example, when providing instructions about the proxy–patient perspective, in some studies, proxies were asked to empathise with the patient, putting themselves in the patient’s shoes [22]. The level of emphasis differed even with in the same perspective category. Some studies added more emphasis, saying, ‘forget yourself” as in a study by Lobchuk et al. [22]. Some studies provide detailed instructions suchas [22] while others for example [14, 46] provide short instructions. One study [30] presented the items in the third person from the proxy patient’s perspective. One study [22] took a neutral perspective, where proxies were not directed or prompted to adopt either of the two perspectives (Table S3).

**Table 3:** Instructions for proxy rating and proxy reporting experience in the included studies

Study	Study type	The instruction used for the proxy–patient version	The instruction used for proxy–proxy version	Proxy reporting experience captured if any	Cognitive interview
Caiels et al. [14]^a^	Validation	Proxies were asked to answer questions from another person’s viewpoint and indicated whether it would be difficult from their own viewpoint	Proxies were asked to answer questions about others from their own viewpoint and report any difficulty	Not reported	Respondents’ comfort level and ease of being a proxy, open-ended opinions on proxy reporting asked qualitatively
Gundy and Aaronson [30]	Dyadic	Proxies were asked to view the situation from the patient’s viewpoint, using third-person statements	Proxies were asked to view the situation from their own perspective	Not reported	N/A
Hong et al. [46]	Dyadic	Proxies were asked to rate how they think their adult child with ASD feels about their own Quality of Life (maternal proxy report)	Proxies were asked to report what they feel about the QoL of their adult child with ASD (maternal report)	Not reported	N/A
Lobchuk et al. [22]^b^	Dyadic	Proxies were asked to imagine how the patient feels and how symptoms affect them. They were asked questions as they believed the patient would	Proxies were asked to imagine how they themselves would feel in the patient’s situation, considering the diagnosis’s impact on their own life	Not reported	N/A
Pickard et al. [47]	Dyadic	Proxies were asked to answer questions as if they were the patient, assessing their health	Proxies were asked to rate patient’s health and abilities from their perspective, which may differ from the patient’s self-view	Not reported	N/A
Rand et al. [48]	Validation	Proxies were asked how they thought the person they represented would answer	Proxies were asked to describe their opinion about the patient’s situation	Not reported	Ability to understand and respond to items based on both perspectives through cognitive interviews
Robertson et al. [49]^c^	Other	DEMQOL-Proxy asked proxies to give answers they thought the patient would provide (proxy–patient perspective)	N/A—only proxy–patient perspective in DEMQOL	Not reported	N/A
Robertson et al. [50]^c^	Other	DEMQOL-Proxy asked proxies to give answers they thought the patient would provide (proxy–patient perspective)	N/A—only proxy–patient perspective in DEMQOL	Not reported	N/A
Silarova et al. [51]	Validation	Proxies were asked to answer how they thought the person they represented would respond	Proxies were asked to answer what they think. A comments box allowed additional input	Not reported	Not reported
Smith et al. [52]	Validation	Proxies were asked to describe how their relative felt in the last week and answered as they believed their relative would	N/A—only proxy–patient perspective in DEMQOL	Not reported	Not reported
Stephan et al. [53]	Validation	Respondents asked to report as if they were the person living with dementia (substituted judgment)	N/A – only proxy–patient perspective in DEMQOL	Not reported	Confidence in responding and understanding each item through cognitive interviews
Tol et al. [32]^b^	Dyadic	Proxies were asked to rate how they think the patient would assess their own health if able	Proxies were asked to rate the health of the patient	Not reported	N/A

^a^This was a qualitative study where participants were asked about their views on both versions of the instructions

^b^They also had a third approach where carers were not explicitly directed to adopt any specific perspective other: neither validation nor dyadic study

^c^While the the 2020 study focuses on quantitatively comparing QoL ratings from staff and family members, the 2019 study qualitatively explores the reasons behind the differences in these ratings. The data sources for the two studies are similar for the qualitative part

#### Perspective adopted by informal carers (2nd objective)

We did not find any discussion in any of the studies as to whether proxies adopted or followed to the instructions provided to them. There was no information regarding the use of any mechanism to check whether the participants had followed the intended perspectives.

#### Proxies’ reporting experience (3rd objective)

Proxies’ reporting experiences were not captured in any of the studies. However, in the validation studies, proxies were asked to reflect on their understanding of the questions through cognitive interviews and open-ended questions. They were asked about how comfortable they were with the questions and the challenges of being a proxy [14, 48], how they interpret the domains in the proxy measures [48, 53], and their confidence in responding [53].

In six of the studies [14, 32, 46–48, 51], the same participants were asked to respond from both perspectives. This means that these participants provided quality of life and symptom measures from both their own perspective and the patient’s perspective simultaneously. In two other studies [22, 30], participants were randomly assigned to respond from one of the two perspectives first and then provided with the other perspective later. This approach involved different subsets of participants responding from either their own perspective or the patient’s perspective initially, and then switching to the other perspective. Additionally, in three studies [49, 50, 53], the instructions for proxies were embedded within the quality of life tool itself. This means that the tool included built-in instructions on how proxies should respond. In contrast, in nine studies, the instructions were specifically developed by the authors for the purpose of the study. These instructions were created to guide proxies on how to respond from the intended perspective. For validation studies, this is particularly the case during the tool’s initial validation study. For example, the DEMQOL tool initially had instructions developed by researchers for its first validation study. Once these instructions were established, they were embedded within the tool for future use. For tools with embedded instructions, researchers have limited flexibility to modify how proxies are instructed to respond from either perspective (Table 4).

**Table 4:** Detailed analysis of perspective instructions

Study	Same sample asked from both perspectives simultanously	Sub sample of the full sample asked from either of the perspectives one after the other	Proxies randomly assigned to either of the perspectives	Proxy instruction is embedded in the quality-of-life tool	Proxy instructions developed for the purpose of the study
Caiels et al. [14]	Y	N	N/A	N	Y
Gundy and Aaronson [30]	N	Y	Y	N	Y
Hong et al. [46]	Y	N	N/A	N	Y
Lobchuk et al. [22]	N	Y	Y	N	Y
Pickard et al. [47]	Y	N	N/A	N	Y
Rand et al. [48]	Y	N	N/A	N	Y
Robertson et al. [49]	N/A	N/A	N/A	Y	N
Robertson et al. [50]	N/A	N/A	N/A	Y	N
Silarova et al. [51]	Y	N	N/A	N	Y
Smith et al. [52]	N/A	N/A	N/A	N	Y
Stephan et al. [53]	N/A	N/A	N/A	Y	N
Tol et al. [32]	Y	N	N/A	N	Y

‘Y’ indicates ‘Yes’, ‘N’ indicates ‘No’, and ‘N/A’ indicates ‘Not applicable’

## Discussion

We conducted a scoping review of the perspective instructions provided to proxies, the extent to which these perspectives were adopted by proxies, and the proxies’ reporting experiences. In this review we identified variation in the instructions given to proxies assessing patient health, and did not find any assessment of whether proxies followed these instructions.

Most of the included studies in the current review involved informal carers of people living with dementia. This is expected, as proxy reporting is prevalent in this population [55] due to the under-development of self-report measures that work for this population [56–58]. The instructions given to proxies in these studies were heterogeneous. In eight of the twelve studies, proxies were asked to report from both perspectives, while in four studies, they were requested to report only from the proxy–patient perspective. Even within the same proxy perspective, variation in the emphasis on perspective taking instructions provided was noted. For example, in a study [22] detailed instruction was provided as follows: “Sometimes when people try to understand what the other person is feeling or thinking, they imagine how they themselves would feel in the person’s situation. We would like you to try the same…While you are doing so, please try to imagine how you would feel if you had the patient’s diagnosis and how this would affect your life. In your mind’s eye, try to picture how you yourself would feel if you were experiencing the same symptoms. Focus on yourself. As you imagine how you would feel if you were diagnosed with the patient’s disease, please tell us…” In contrast, in another study [47] the instruction for proxy–patient perspective was: “We are interested in how you think the patient would assess their health. Answer these questions as if you are the patient.” Providing consistent instructions on perspective-taking may help proxies better understand how to approach the questions they are asked. This, in turn, could result in more interpretable and useful data. Specifically, consistent instructions can enhance the reliability of data collected by proxies, making it easier to observe changes over successive appointments.

Additionally, consistency between different proxy reporters can ensure that the data is comparable and interpretable, leading to more accurate assessments of quality of life [25]. The heterogeneity of proxy instructions observed in our study aligns with a previous systematic review on proxy ratings for patients with primary brain tumors [59]. Studies have emphasised the importance of clearly defining and documenting the proxy perspective [60, 61]. Reviews have also indicated that clear instructions should be provided to proxy respondents [7, 62]. This clarity may be helpful for healthcare providers, significant others, and proxy respondents when evaluating patients’ quality of life [63]. Clear instructions help proxies respond from the intended perspective [61]. Providing detailed guidance in instructions helps clarify whether proxies should respond based on their own views or those of the care recipient [14]. These studies collectively reinforce the importance of providing proxies with clear, well-defined instructions to collect better quality data.

In addition to providing perspective instructions, it might be important to check whether proxies understand and follow instructions as proxies might not always adhere to the instructions provided. Researchers should ensure that proxies understand their role and the intended perspective (their own or the care recipient’s), as this understanding is crucial for accurate data interpretation and reliable results. Evaluating content validity of the instructions, clear definition of intended perspective as suggested by previous guidance [61]. Asking proxies about their reporting experience and any challenges they encountered at the end of the survey may provide helpful context to responses.

The included studies did not specifically examine proxies’ reporting experiences. However, insights from validation studies using cognitive interviews and open-ended questions have shed light on proxies’ comfort, confidence, and difficulties in responding. During these cognitive interviews, proxies were asked about their interpretations of the domains in the proxy measures. By incorporating closed-ended probing questions that assess the ease or difficulty and confidence level in answering specific survey questions, researchers can evaluate whether proxies’ responses to these questions are linked to data quality. A higher perceived ease or confidence may suggest a greater likelihood of accurate matches between proxy and self-responses [36]. Researchers may wish to include debriefing questions at the end of the surveys to assess respondents’ experience of completing the survey. The debriefing questions may  help in assessing  respondents' percieved difficulty while  completing questionnaires [64]. This information can provide valuable context for interpreting the findings. When proxies are not given clear instructions regarding the perspective they need to adopt, it may lead to inconsistent data and make interpretation of results difficult as it will not be clear whether the respondents follow their own or the patients’ perspective.

## Limitation and strength of the review

One limitation of this review is the exclusion of grey literature, which may have led to relevant studies being overlooked. For example, relevant reports or theses could contribute to a greater understanding of the topic. Additionally, as most studies in the review were in dementia and cancer populations, it is not clear how generalisable these findings are to other conditions. The small number of included studies potentially limits the strength of the evidence but also underscores an important research gap. Specifically, relatively little is known about proxies, despite their important role in providing data in many situations. This lack of information makes it difficult to improve questionnaire design. Additional work can help address this gap.

Despite these limitations, the use of a systematic search strategy, comprehensive literature review, double screening, and rigorous methodology—including predefined eligibility criteria and adherence to established JBI guidelines—enhances the credibility and robustness of the review’s findings. These methodological strengths provide a comprehensive overview of the current state of research on proxy reporting instructions for informal carers, ensuring that the conclusions drawn are well-supported and reliable.

## Conclusions

The review highlights the need for more consistency in instructions given to proxies, as well as information on how well proxies follow these instructions and their experience of reporting. This can help improve questionnaire design and the quality of proxy-reported information about the health of others. Providing clear instructions helps the researchers and clinicians to collect useful and interpretable information from informal carers. To this end, clear instructions and an understanding how and if instructions are followed and interpreted can be useful in interpreting proxy-reported information.

## Supplementary Information

Below is the link to the electronic supplementary material.

Supplementary file1 (DOCX 79 KB)

## Data Availability

All the supporting data is included along with the manuscript. Details of the review protocol can be found on Open Science Framework https://osf.io/357r4.

## Abbreviations

ASCOT: Adult Social Care Outcomes Toolkit
EORTC QLQ-C30: European Organisation for Research and Treatment of Cancer Quality of Life Questionnaire-Core 30
WHOQOL-BREF: World Health Organization Quality of Life-BREF
ICECAP-A: ICEpop CAPability measure for Adults
MSAS (shortened version): Memorial Symptom Assessment Scale (shortened version)
EQ-5D: EuroQol 5-Dimension Questionnaire
DEMQOL-Proxy: Dementia Quality of Life Proxy instrument
ICECAP-O: ICEpop CAPability measure for Older people
EQ-5D-5L: EuroQol 5-Dimension 5-Level Questionnaire
QOL-ACC: Quality of Life-Aged Care Consumers
