# Supplementary material for: Proxy reporting in health: a scoping review of instructions, perspectives, and reporting experiences
Source: Qual Life Res. 2025 Feb 26;34(7):1835–47. doi: 10.1007/s11136-025-03929-8 (PMC12182471; doi:10.1007/s11136-025-03929-8)
Supplement: Supplementary file 1 — Supplementary file1 (DOCX 79 KB) [file 11136_2025_3929_MOESM1_ESM.docx]

**Supplementary File 1: search strategy**

**Medline via ovid, Embase and Psych Info**

**Proxy term**

1. (Proxy adj3 report*).mp.
2. (Proxies adj3 report*).mp.
3. (External* rat* adj3 report*).mp.
4. (care* adj3 report*).mp.
5. (famil* adj3 report*).mp.
6. ('next of kin' adj3 report*).mp.
7. (husband adj3 report*).mp.
8. (wife adj3 report*).mp.
9. (partner adj3 report*).mp.
10. (surrogate adj 3 report*).mp
11. Proxy rating
12. Proxy version
13. Proxy perspective
14. Proxy-patient
15. Proxy-proxy

**Quality of life term**

1. quality of life/
2. QOL.mp.
3. QL.mp.
4. HRQL.mp.
5. HRQOL.mp.
6. outcome* assessment.mp.
7. symptom* assessment.mp.
8. quality of life.mp.
9. health status/
10. health status.mp.
11. ((social or physical or role or emotional or cognitive) adj1 function*).mp.
12. wellbeing.mp.
13. patient status.mp.

**Exclusion**

1. exp infant/
2. exp child/
3. p?ediatric.mp.

Boolean terms

1. OR/1-15
2. OR/ 16-28
3. 32 and 33
4. OR/29-31
5. 34 not 35
6. limited 36 to (English Language and humans)

CINAHL

(Proxy adj3 report*).mp. OR (Proxies adj3 report*).mp. OR (External* rat* adj3 report*).mp. OR (care* adj3 report*).mp. OR (famil* adj3 report*).mp. OR ('next of kin' adj3 report*).mp. OR (wife adj3 report*).mp. OR (partner adj3 report*).mp. OR (surrogate adj 3 report*).mp OR Proxy rating OR Proxy version OR Proxy perspective) AND All adults

Date searched: We ran the search on 13/07/2023 for all four databases. The date of coverage for each of the databases was as described below.

- Medline via Ovid 1946 -Present
- Embase: 1974 to present
- PsycINFO: 1806 to present
- CINAHL: 1993 to present

Table S1: Eligibility criteria for study inclusion before protocol amendment

| Criteria | Inclusion | Exclusion |
| --- | --- | --- |
| Population | Informal carers of adults only | - Formal carers - Carers acting on care plan and proxy decision making, - Carers acting as agent in care plan |
| Concept | - Proxy instructions, - Proxy instruction adherence and - Proxies’ experience. | - Concepts other than the three mentioned ones. |
| Study type | - Original articles reporting on either of the three concepts. - Validation studies if any proxy instruction - Concordance studies with multiple perspectives - Articles written in English | - Articles that do not include any of the three concepts. - Validation studies with no information about perspectives - Articles written in language other than English - Grey literature - Dyadic studies |
| Time | Until 13/07/2023 (no starting date and no country-related limitations were applied), |  |
| Study design | Primary studies with any design |  |
| Information sources | Medline via Ovid, Psych Info, CINAHL, and Embase. |  |
| Key terms | **Proxy terms:** proxy report, external rater report, carer report, family report  **Perspective:** proxy version, proxy perspective, proxy-patient, proxy-proxy  **Quality of life term:** QOL, HRQL, HRQOL |  |

**Table S2: aims of studies included in the review**

| **Authors, year** | **Aims** |
| --- | --- |
| Caiels et al. 2019 | Exploring the views of being a proxy from the perspective of unpaid carers and paid carers: developing a proxy version of the Adult Social Care Outcomes Toolkit |
| Gundy and Aaronson 2008 | To evaluate the influence of proxy perspective on patient-proxy agreement in the evaluation of health-related quality of life |
| Hong et al. 2016 | To assess factors associated with subjective quality of life of adults with autism spectrum disorder: Self-Report Versus Maternal Reports |
| Lobchuk et al. 2007 | To test if the interrater gap between patient self-assessment and caregiver assessment on patient multidimensional symptom experiences will be reduced when caregivers are prompted to imagine-patient perspective-take. |
| Pickard et al. 2009 | To determine if informal caregiver assessments of patients with prostate cancer differed when prompted from both the patient perspective (proxy-patient) and their own viewpoint (proxy-proxy), and to identify factors associated with differences in proxy perspectives. |
| Rand et al. 2017 | To evaluate the acceptability and content validity of the ASCOT-Proxy |
| Robertson et al. 2019 | To explore why staff and family think differently about quality of life |
| Robertson et al. 2020 | Comparing proxy-rated quality of life of people living with dementia in care homes |
| Silarova et al. 2023 | To establish the feasibility, construct validity and reliability of the ASCOT-Proxy and ASCOT-Carer, with unpaid carers of people with dementia living at home unable to self-report. |
| Smith et al. 2005 | To develop a conceptual framework of HRQL in dementia from the perspective of people with dementia and their carers and to examine differences in the reports of the HRQL of these two groups. |
| Stephan et al. 2021 | Linguistic validation of ICECAP-O self and proxy report |
| Tol et al. 2021 | To determine the level of agreement between both proxy versions and the self-completed EQ-5D-5L |

Table S3: Instructions for proxy rating and proxy reporting experience in the included studies

| **Study** | **The instruction used for the proxy-patient version** | **The instruction used for proxy-proxy version** |
| --- | --- | --- |
| Caiels et al. 2019 | “Would you prefer to answer it from the viewpoint of the person you support? Would it be difficult to answer from their viewpoint?” | “Would you prefer to answer it from your viewpoint?” |
| Gundy and Aaronson 2008 | Authors mentioned standard instructions were provided in which proxies were asked to try to view the situation from the patients’ perspective. All item statements were made from the third-person perspective (eg, “Would the patient say that he/she...”). | The proxies were instructed to view the situation from their own, personal perspective, and to complete the questionnaire accordingly. |
| Hong et al. 2016 | How she feels about the QoL of her adult child with ASD (maternal report) | How she thinks her adult child with ASD feels about his/her own QoL (maternal proxy-report) |
| Lobchuk et al. 2007 | "Sometimes when people try to understand what the other person is feeling or thinking, they imagine how they themselves would feel in the person’s situation. We would like you to try the same for the six symptoms listed below. Read each question carefully. While you are doing so, please try to imagine how you would feel if you had the patient’s diagnosis and how this would affect your life. In your mind’s eye, try to picture how you yourself would feel if you were experiencing the same symptoms. Focus on yourself. As you imagine how you would feel if you were diagnosed with the patient’s disease, please tell us how often you would have the symptom, how severe the symptom would be, and how much it would distress or bother you by circling the appropriate number. If you do not believe you would have the symptom, make an ‘X’ in the box marked would not have." | "We have listed six symptoms below. Read each question carefully. While you are doing so, please try to imagine how the patient feels and how these symptoms are affecting him or her. In your mind’s eye, put yourself in the patient’s shoes. Forget yourself. Try to picture how the symptom feels to the patient. Answer the questions as you believe the patient would. Please tell us how often the patient would say he or she had the symptom, how severe the patient would say the symptom was usually, and how much distress or bother the patient would say the symptom caused him or her by circling the appropriate number. If the patient would say he or she did not have the symptom, make an ‘X’ in the box marked did not have" |
| Pickard et al. 2009 | “We are interested in how you think the patient would assess their health. Answer these questions as if you are the patient.” | "We are interested in the patient's health and ability/capabilities from your perspective, which may or may not be the same as how the patient might view themselves"​ |
| Rand et al. 2017 | “Which of the following statements best describes how the person you represent spends his/her time.? Please say how you think the person you are representing would answer in the second column.” | “Which of the following statements best describes how the person you represent spends his/her time? Please say what you think in the first column.” |
| Robertson et al. 2019 | DEMQOL-Proxy asked proxies to give answers they thought the patient would provide (proxy-patient perspective). | N/A– only proxy-patient perspective in DEMQOL |
| Robertson et al. 2020 | “I would like to ask you about _________ (your relative’s) life, as you are the person who knows him/her best. There are no right or wrong answers. Just give the answer that best describes how _________ (your relative) has felt in the last week. If possible try and give the answer that you think _________ (your relative) would give. Don’t worry if some questions appear not to apply to _________ (your relative). We have to ask the same questions of everybody.” | N/A – only proxy-patient perspective in DEMQOL |
| Silarova et al. 2023 | "Please say how you think the person you are representing would answer Please write in the comments box if you wish to add anything to your answer. " | "Please say what you think . Please write in the comments box if you wish to add anything to your answer. " |
| Smith et al. 2005 | “I would like to ask you about _________ (your relative’s) life, as you are the person who knows him/her best. Just give the answer that best describes how _________  (your relative) has felt in the last week. If possible try and give the answer that you think _________ (your relative) would give. Don’t worry if some questions appear not to apply to _________ (your relative). We have to ask the same questions of everybody. Before we start we’ll do a practice question; that’s one that doesn’t count. (Show the response card and ask respondent to say or point to the answer.) In the last week how much has _________ (your relative) enjoyed watching television?” | N/A – only proxy-patient perspective in DEMQOL |
| Stephan et al. 2021 | Respondents asked to report as if they were the person with dementia (substituted judgment). | N/A – only proxy-patient perspective in DEMQOL |
| Tol et al. 2021 | “How do you think the patient would rate their own health if they were able to do so” | “How do you rate the patients’ health in your opinion” |
|  | | |
